# The mast cell/S1P axis is not linked to pre-lesional male skin remodeling in a mouse model of eczema

**DOI:** 10.3934/allergy.2021012

**Published:** 2021-05-28

**Authors:** Ross M. Tanis, Piper A. Wedman-Robida, Alena P. Chumanevich, John W. Fuseler, Carole A. Oskeritzian

**Affiliations:** 1 Department of Pathology, Microbiology and Immunology, University of South Carolina School of Medicine, Columbia, SC 29209, USA; 2 Department of Internal Medicine, Loyola University Medical Center, 2160 South 1st Avenue, Maywood, IL 60153, USA; 3 Department of Natural Science, Northwestern Oklahoma State University, Science Building 100-D, 709 Oklahoma Boulevard, Alva, OK 73717, USA

**Keywords:** pathogenesis, mast cells, sphingosine-1-phosphate, chemokines, animal models, eczema

## Abstract

Atopic dermatitis (AD, eczema) is an inflammatory skin condition whose histopathology involves remodeling. Few preclinical AD studies are performed using male mice. The histopathological mechanisms underlying AD development were investigated here in male mice at a pre-lesional stage using a human AD-like mouse model. Hypodermal cellular infiltration without thickening of skin layers was observed after one epicutaneous exposure to antigen ovalbumin (OVA), compared to controls. In contrast to our previous report using female mice, OVA treatment did not activate skin mast cells (MC) or elevate sphingosine-1-phosphate (S1P) levels while increasing systemic but not local levels of CCL2, CCL3 and CCL5 chemokines. In contrast to the pathogenic AD mechanisms we recently uncovered in female, S1P-mediated skin MC activation with subsequent local chemokine production is not observed in male mice, supporting sex differences in pre-lesional stages of AD. We are proposing that differential involvement of the MC/S1P axis in early pathogenic skin changes contributes to the well documented yet still incompletely understood sex-dimorphic susceptibility to AD in humans.

## 1. Introduction

Atopic dermatitis (AD) affects nearly 31.6 million (10.1%) people in the United States [1] and of these, 18 million adults (7.2%) over the age of 18 have AD [2]. Gender disparities have been noted in the prevalence of AD with females being more prone during adulthood [3-12]. Data is lacking in males that supports mechanistic differences between the two sexes. Thus, many preclinical AD studies utilize female mice [13-14], with rare exceptions [14,15].

The pathophysiology of AD involves epidermal barrier dysfunction, a Th2/Th22 helper cell immune response and genetic polymorphisms which have a more uncertain role [16,17]. Current therapies for AD are developed for lesional skin, although non-lesional skin is perturbed [17]. We previously reported pre-lesional skin remodeling and increased activation of mast cells (MC), skin-resident innate immune cells, via the signaling of a potently bioactive sphingolipid metabolite, sphingosine-1-phosphate (S1P), observed in female mice after 1 week of OVA treatment [18,19]. This novel finding uncovered histologic and biochemical perturbations occurring in the skin before the development of any overt lesions. Here, we report pre-lesional skin responses in male mice upon a similar short 7-day epicutaneous exposure to OVA. We found that, conversely to female mice [18,19], males did not exhibit any pre-lesional disease-inducing skin remodeling or MC activation. Importantly, samples from our previous report on female mice [18] and from the current report on male mice were collected and analyzed at the same time, following a gender-matched experimental design. While we did not ascertain the mechanism leading to the observed gender disparity, we established histological and biochemical differences in OVA-exposed male mouse skin compared to females that may partly elucidate the sex differences substantiated in adult AD prevalence.

## 2. Materials and methods

### 2.1. Atopic dermatitis model

AD was induced in 8 to 12 weeks old male C57Bl/6J (WT) mice exactly as previously described and at the same time as the female mouse study [18,20] (Charles River NCI, Frederick, MD, USA and The Jackson Laboratory Bar Harbor, ME, USA). Mouse randomization was performed using shuffled pieces of paper indicating treatment to assign mice to either experimental group. One by one centimeter square-sized gauze pad (“patch”) containing one hundred microliters of solution of either 100 μg OVA (Sigma-Aldrich, St Louis, MO, USA) or 0.9% saline vehicle only (saline control) was positioned on the shaved dorsal skin area after tape stripping. Patches were secured in position for seven days using a transparent dressing (Tegaderm, 3M Healthcare, St Paul, MN, USA) and bandages. Treated skin areas were harvested from euthanized mice. The AD model was validated as previously (our results [18] and [20]) through serum IgE level quantification in male mice that received three epicutaneous exposures, performed at the same time as in female mice. The full AD protocol consists in three rounds of one seven-day skin exposure via patch followed by fourteen days without patch. Circulating IgE levels were measured in sera from blood samples collected by cardiac puncture from euthanized mice and kept at −80 °C until use.

### 2.2. Ethics approval of research

All protocols were approved by the University of South Carolina Institutional Animal Care and Use Committee (Protocol 2403-101303-010818, “Mast cells and sphingosine-1-phosphate in allergic inflammation”). All experiments were performed in accordance with guidelines and regulations.

### 2.3. Histopathological analysis

Paraffin-embedded skin tissues were fixed with fresh paraformaldehyde (4%) and cut into 4 μm sections. Some sections were hematoxylin and eosin (H&E)-stained for morphometric analysis as previously described [18]. Others were stained with 0.1% methylene blue (MB) for 5 seconds, rinsed with water, dehydrated, and coverslip-mounted to locate MC and MC activation status [18,21,22]. All imaging and measurements were performed by blinded investigators. Imaging was performed at 10× and 40× magnifications using a Nikon E-600 microscope (Nikon Inc., Melville, NY) and a Micropublisher digital camera model 5.0. The QImaging software version 2.0.13 (QImaging Corp., Surrey, BC, Canada) was utilized for image analysis of 24-bit color or 8-bit monochrome images. The MetaMorph^®^ 6.1 software (Molecular Devices, Sunnyvale, CA, USA) and the HarFa imaging software (www.fch.vutbr.cz/lectures/imagesci/) were employed to calculate morphometric and fractal parameters of nuclei allowing for infiltration quantification and measure MC status. Infiltration was measured in 25 to 30 ROIs per image using at least twelve 40× images per skin section and 4 skin sections per 2 mice from each treatment group. MC activation status (percent degranulation) was calculated *in situ* after analyzing fifty 40× images and 310 to 485 total MC present in 4 skin sections from 2 mice in each treatment group [18,22].

### 2.4. Skin layer measurements

Epidermis, dermis, and hypodermis thickness was measured in saline- and OVA-treated samples with an ocular micrometer (Klarmann Rulings, Inc., Litchfield, NH, USA), exactly as described [18,22]. The average thickness was calculated from 3 to 5 measurements (n = 3–5 mice per treatment group for each skin layer).

### 2.5. Computer-aided measurement of cellular infiltration

We adapted our innovative imaging methods [22] to measure hypodermal cellular infiltration [18], using at least twelve high-magnification H&E images per individual mouse. First, the set-color-threshold subroutine (MetaMorph software) was used to isolate nuclei by color thresholding [18]. Next, the hypodermis in each section was surveyed using a fixed 75 μm diameter-sized region of interest (ROI) [18]. Thus, 25 to 30 ROIs were evaluated per skin section for a total of 2 sections per slide, using 2 slides for each individual mouse and 2 mice for each treatment group. Our previous work validated the morphometric parameters of area, perimeter, and integrated optical density (MetaMorph software’s Integrated Morphometry Analysis subroutine) for accurate quantification of nuclei with elimination of nonspecific staining [18].

### 2.6. Independent confirmation of infiltration quantification using fractal dimension analysis

After thresholding (Section 2.5), fractal dimensions (D) were measured after 8-bit grayscale image conversion exactly as previously described [18,22].

### 2.7. Quantification of mast cell degranulation in skin tissue samples

We established an imaging method to quantitatively described the cytoplasm of MC using fractal geometry [22], demonstrating that a fractal dimension (D) value of 1.378 ± 0.062 specifically designated intact/resting MC, whereas a D value of 1.484 ± 0.048 characterized degranulated/activated MC [22]. Here, we used these D values for nonsubjective measure of MC activation in male skin tissue samples (n = 2 to 5 mice per treatment group).

### 2.8. Quantitative real-time polymerase chain reaction (qPCR)

Harvested skin tissue samples were immediately snap-frozen, and kept at −80 °C until use. Total RNA was extracted from 4 to 10 mice per treatment group using the miRNeasy kit (Qiagen, Valencia, CA, USA) and cDNA synthesized by reverse transcription (iScript cDNA synthesis kit, Bio-Rad, Hercules, CA, USA). A CFX Connect (Bio-Rad) was used to perform qPCR (duplicate determinations), using SensiFAST^™^ SYBR No-ROX Kit (Bioline, Taunton, MA, USA). We used the following primers (all purchased from Thermo Fisher Scientific, Inc., Waltham, MA, USA). for qPCR amplification: GAPDH forward primer CAGAAGGGGCGGAGATGAT and reverse primer AGGCCGGTGCTGCTGAGTATGTC, FcεRIα forward primer ATTGTGAGTGCCACCGTTCA and reverse primer GCAGCCAATCTTGCGTTACA, CCL2 forward primer CACTCACCTGCTGCTACTCA and reverse primer GCTTGGTGACAAAAACTACAGC, CCL3 forward primer GCCATATGGAGCTGACACCC and reverse primer TAGTCAGGAAAATGACACCTGGC, and CCL5 forward primer TGCCCTCACCATCATCCTCACT and reverse primer GGCGGTTCCTTCGAGTGACA. We used the following qPCR conditions: initial step at 95 °C for 5 minutes and 40 cycles, as follows: 10 seconds at 95 °C, followed by annealing for 1 minute at 55 °C and extension for 1 minute at 72 °C. Data were analyzed with CFX Manager^™^ Software, after normalization to control (saline-treated) samples and to the amount of reference gene, GAPDH mRNA levels.

### 2.9. Multiplex chemokine assays

CCL2, CCL3 and CCL5 chemokine quantifications were conducted in mouse serum and whole skin protein extracts prepared exactly as previously described [18], using a Bio-Plex Array Reader (LUMINEX 100; Bio-Rad Laboratories, Hercules, CA, USA) and Milliplex panels (EMD Millipore, Billerica, MA, USA). Whole skin protein extracts were weighed and immediately snap-frozen using liquid nitrogen. After homogenization with a mortar and pestle, skin samples were digested for 2 hours at 4 °C in reporter lysis buffer (Promega, Madison, WI, USA) supplemented with complete mini-protease inhibitors (Roche Diagnostics, Indianapolis, IN, USA). Supernatants were kept at −80 °C until use.

### 2.10. IgE ELISA

Total IgE was quantified in mouse serum by ELISA (R&D Systems, Minneapolis, MN, USA). N = 6 and 9 mice per experimental group for 1 and 3 exposures, respectively.

### 2.11. Lipidomics

Lipids were extracted from snap-frozen skin tissues and S1P or sphingosine was quantified by liquid chromatography-electrospray ionization-tandem mass spectrometry (4000 QTRAP; AB Sciex, Foster City, CA, USA), as before [18], and normalized to skin sample weights. Saline and OVA solutions contained no detectable levels of S1P or sphingosine (data not shown). N = 3 to 4 samples per experimental group.

### 2.12. Statistical analysis

Expressed as means ± SEM (unless otherwise indicated), data was analyzed using the unpaired 2-tailed Student’s *t*-test, with Welch’s correction for samples of unequal variance (Prism 6; GraphPad Software, La Jolla, CA, USA). Significance for all statistical tests is provided in figures. Each experiment was repeated at least 3 times in triplicate determinations with consistent results.

## 3. Results

### 3.1. A single OVA skin exposure leads to cellular infiltration without skin remodeling in male mice

**Figure 1.**
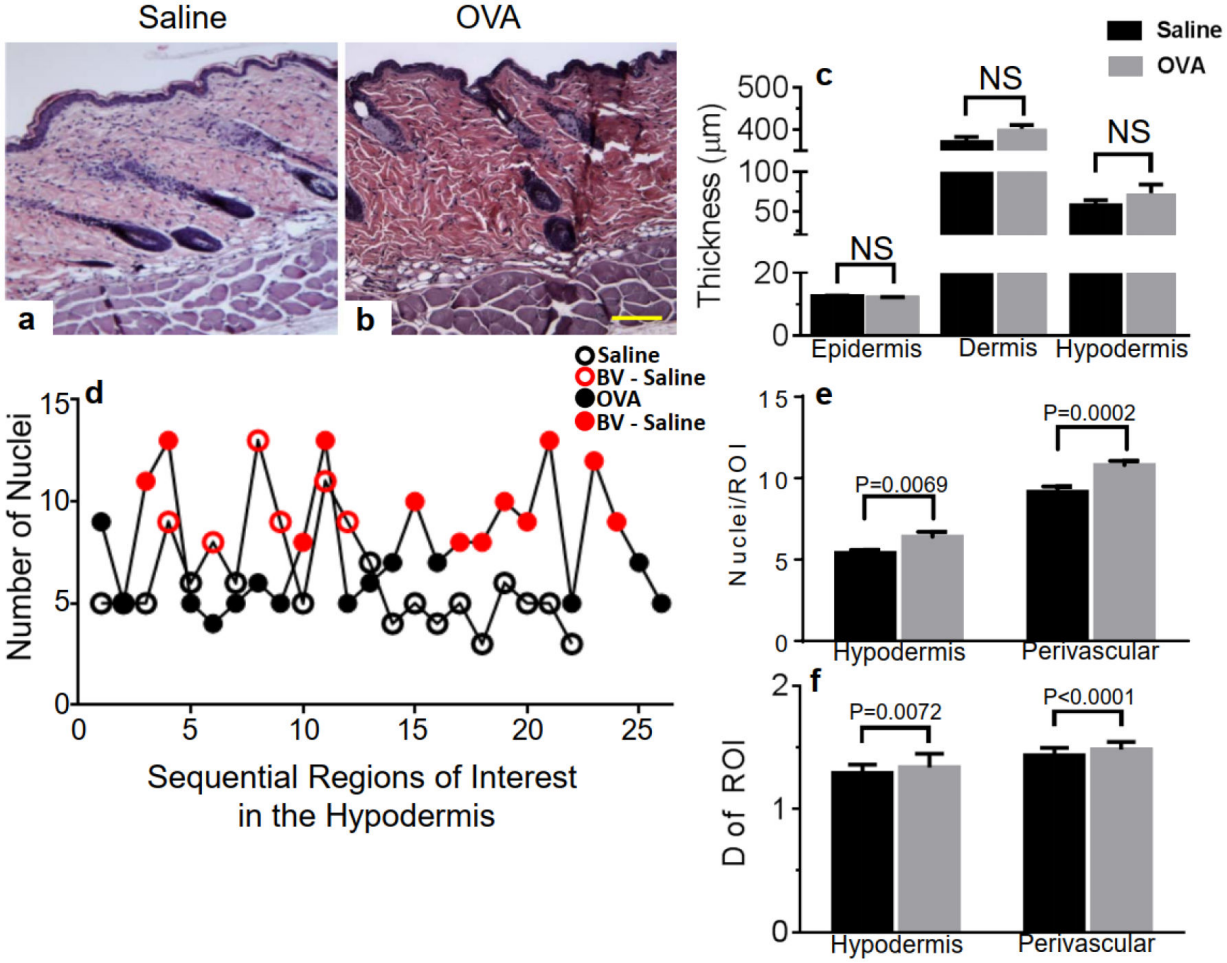
Skin remodeling quantitation in differentially treated male skin samples. Hypodermal cellular infiltration does not cause skin thickening after OVA exposure. Representative images of H&E-stained skin sections of (a) control (b) OVA treated mice. Original magnification 10× (scale bar = 50 μm). (c) Epidermal, dermal and hypodermal thickness. (d) Representative nuclei quantification in each region of interest across the hypodermis of one saline- (empty circles) and one OVA-treated (filled circles) mice, also distinguishing blood vessel (BV)-containing ROI (red symbols). (e) Quantification of cellular infiltration (nuclei per ROI) after saline (black symbols) or OVA (red symbols) skin treatment. (f) Validation of cell quantification by fractal dimension (D) of each ROI. N = 10–20 individual measurements collected from 3 to 5 mice per treatment group and per skin layer. ROI determinations: 25 to 30 ROI per image, at least 12 40×-images per skin section, 4 skin sections, 2 mice per treatment group.

We previously demonstrated that the epidermis and dermis were thickened in female mice after a single dose of OVA applied to the skin [18]. Moreover, there was an increase in cellular infiltration particularly around blood vessels (BV) [18]. Representative images of H&E-stained saline (Figure 1a) and OVA (Figure 1b) exposed male mice skins illustrate that no significant change was evidenced in epidermal, dermal or hypodermal thickness after 1 week of OVA treatment compared to controls (Figure 1c), in contrast to our reported findings in female mice collected at the same time [18]. We then measured cellular infiltration in the hypodermis by color thresholding the nuclei in the entire hypodermis of H&E-stained skin sections, exactly as previously described [18]. We report increased cellular infiltration in the hypodermis and around BV (Figure 1d-e) after OVA exposure of male dorsal skins, compared to saline controls. In order to independently confirm these results, we applied yet another of our previously published computerized imaging modalities to quantify cellular mass, a proxy for cell numbers/infiltration, by means of fractals, or space filling capacity [18,22]. Cellular infiltration of the hypodermis (Figure 1d-e) was confirmed by an increase in cellular mass, expressed as augmented fractal dimension (D) values in the hypodermis and around BV (Figure 1f) after OVA treatment and compared to saline controls.

### 3.2. OVA treatment does not cause elevation of skin MC numbers or degranulation in male mice

**Figure 2.**
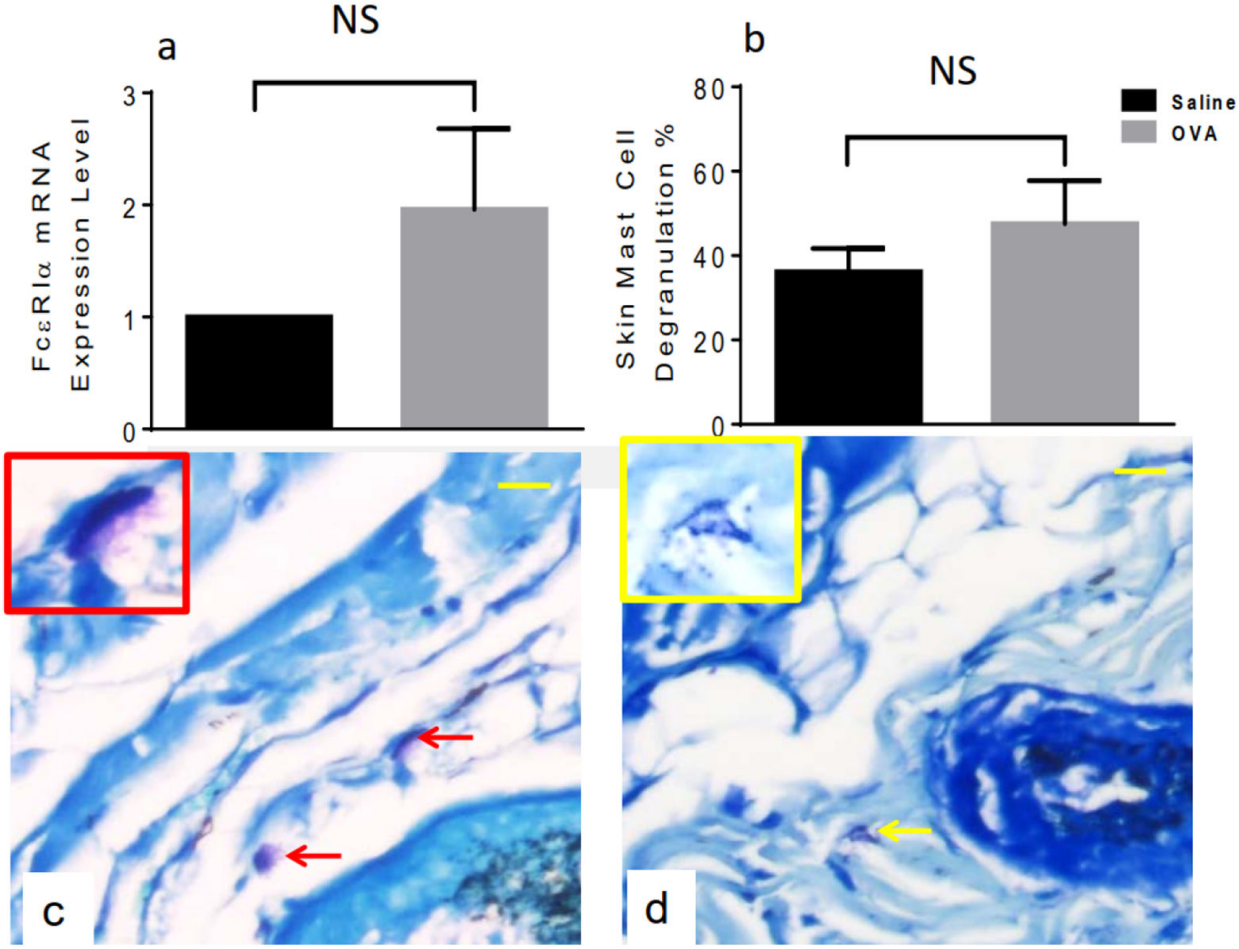
Mast cell activation status in differentially treated male skin samples. OVA exposure does not cause skin MC activation after one OVA exposure in male mice. FcεRIα (a) and percentage of degranulated MC (b) after OVA treatment (grey columns) compared to saline controls (black columns). Representative images of methylene blue-stained skin sections collected after saline (c) or OVA (d) exposure. Arrows indicate intact (red) and degranulated (yellow) MC, as detected by a validated computer-aided image analysis method. The insets represent magnifications of resting (red frame) or a degranulated (yellow frame) MC at 40×. Bar = 50 μm. Unpaired 2-tailed Student’s *t*-test with Welch’s correction. NS, not significant.

We previously reported that mRNA levels coding for FcεRIα, the IgE-binding sub-unit of the high-affinity receptor for IgE reflected local MC numbers in this animal model of AD [18], yet were not significantly increased after OVA treatment, neither was MC degranulation (Figure 2b,d) compared to saline controls (Figure 2a,c) and in contrast to female mice [18].

### 3.3. The MC-activating S1P/SphK1 axis is not induced in male mice skin upon one OVA application

**Figure 3.**
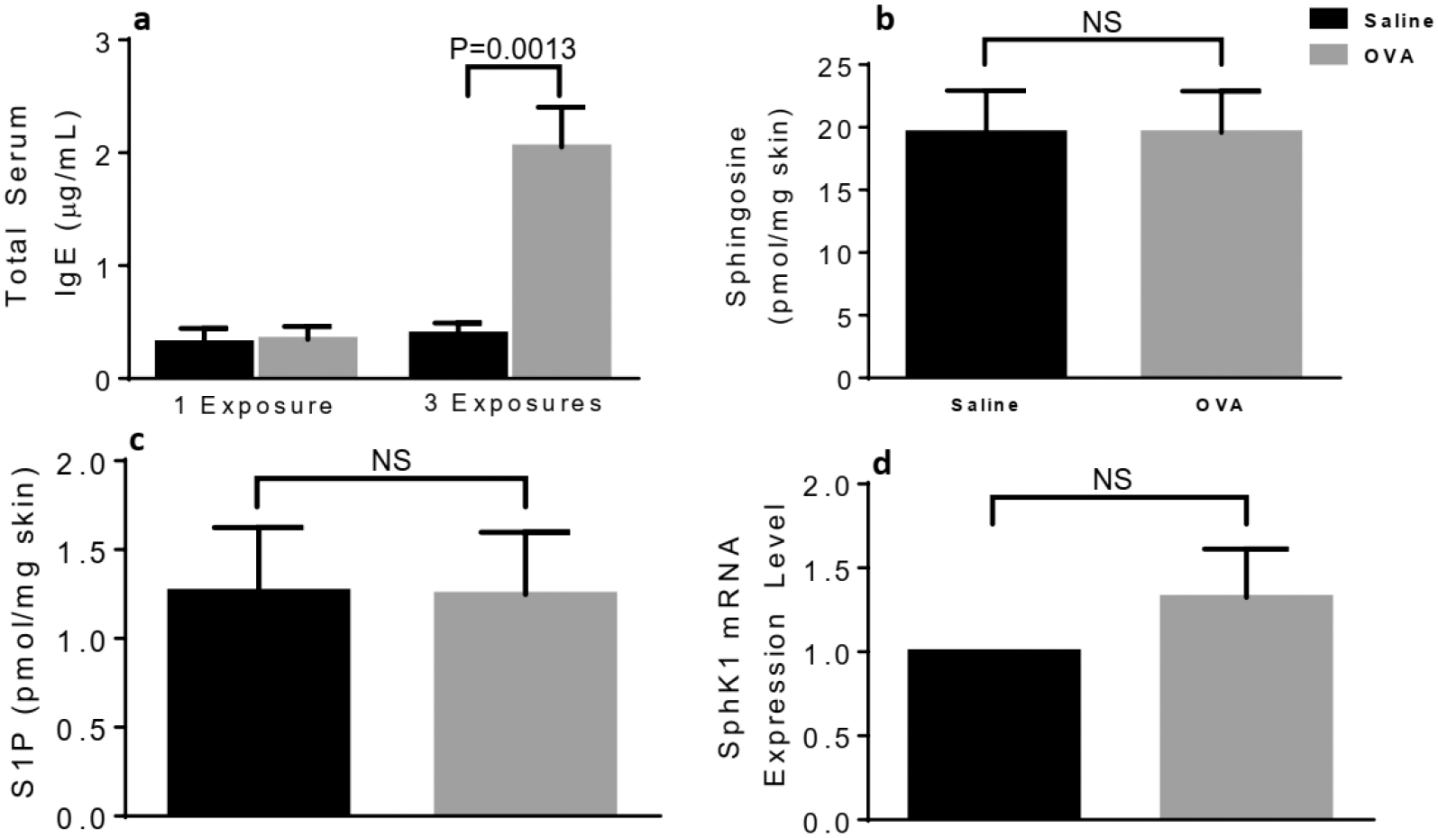
Circulating IgE levels, skin-associated sphingolipid sphingosine and S1P and SphK1 mRNA levels in differentially treated male skin samples. One OVA exposure does not elevate skin sphingosine or S1P levels, SphK1 mRNA expression or circulating IgE levels in male mice. (a) Serum IgE levels after 3 OVA or saline treatments. Skin levels of sphingosine (b), S1P (c) and SphK1 mRNA expression (d). Unpaired 2-tailed Student’s *t*-test with Welch’s correction. NS, not significant.

As expected, the serum levels of IgE were not increased after one single OVA exposure but were increased after 3 OVA exposures (Figure 3a). Thus, we previously established that skin MC degranulation at this early stage of AD was not due to the classical IgE/Ag activation pathway but rather depended on S1P signaling in MC [18,19]. Unaltered MC degranulation in male skin was concurrent to unchanged local levels of sphingolipid metabolites sphingosine (Figure 3b) and S1P (Figure 3c) and Sphk1 mRNA after OVA treatment compared to saline controls (Figure 3d), confirming the newly identified mechanistic insights we reported that pertained to AD onset [18,19].

### 3.4. Serum not local chemokines are elevated in OVA treated male mice

**Figure 4.**
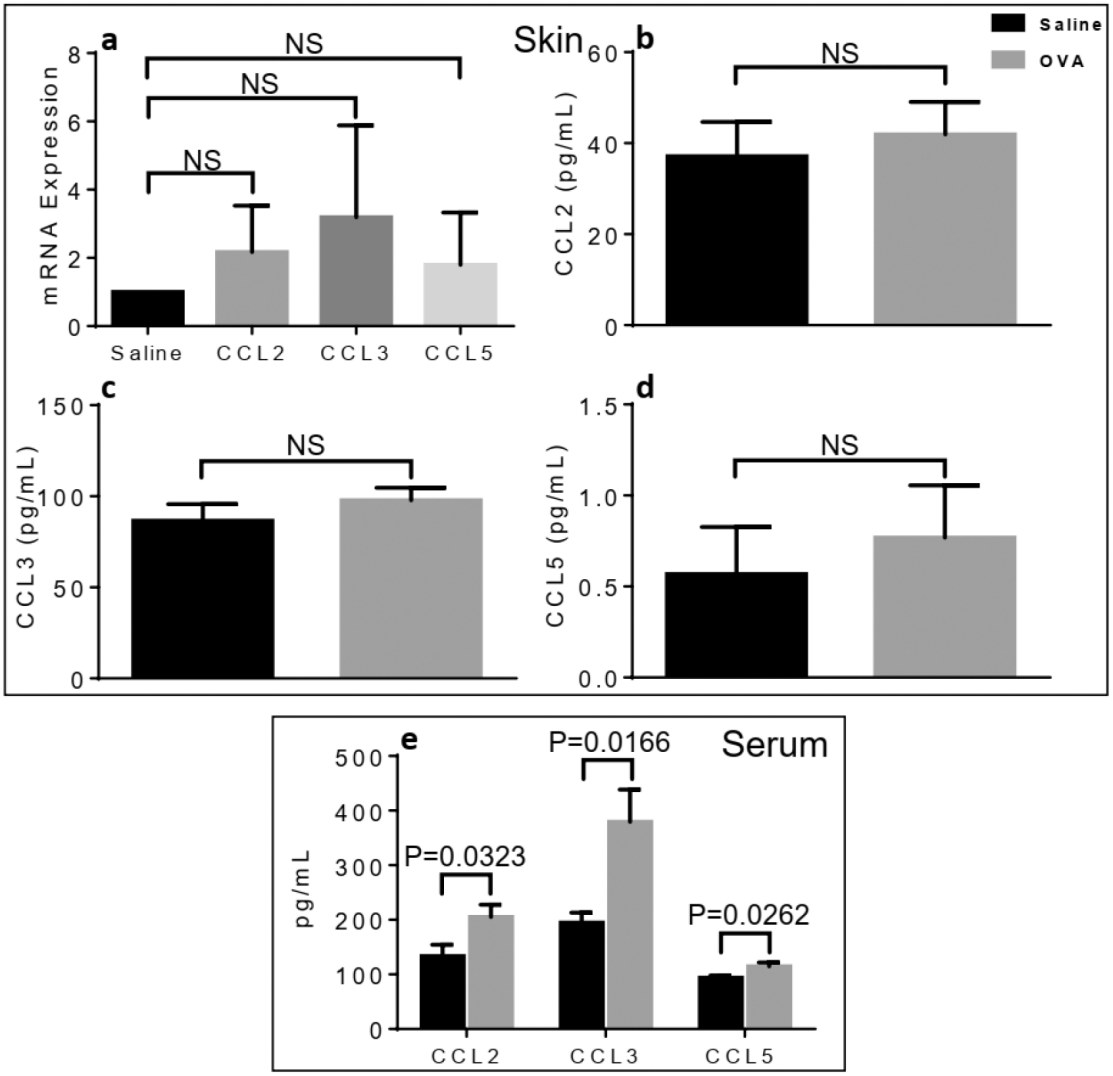
Skin-associated *versus* circulating levels of chemokines in differentially treated male mice. Systemic but not local chemokines are elevated after one OVA exposure in male mice. (a) Skin CCL2, CCL3 and CCL5 mRNA expression. Skin protein levels of CCL2 (b), CCL3 (c) and CCL5 (d) and their circulating levels (e) in OVA- or saline-treated mice. Unpaired 2-tailed Student’s *t*-test with Welch’s correction. NS, not significant.

The elevation of local cytokines/chemokines drives the development of AD, whereas their systemic augmentation correlates with disease progression and/or severity [17], We previously demonstrated that airway [23] and skin [18] inflammatory responses were linked to local rather than systemic elevation of chemokines, thus demonstrating their pathogenic function in atopy. Whereas local levels of CCL2 (Figure 4a,b), CCL3 (Figure 4a,c) and CCL5 (Figure 4a,d) chemokines did not augment upon OVA skin treatment, circulating CCL2, CCL3 and CCL5 were significantly elevated in OVA-exposed male mice skin, compared to saline controls (Figure 4e).

## 4. Discussion and conclusions

Female mice are predominantly used in established preclinical models of AD that encompass epicutaneous applications of OVA [20], partly because they feature stronger antigen-dependent cutaneous inflammatory responses than males, similar to the female preponderance of AD observed in adolescent and adult humans [12,24-26]. Although no single murine model fully recapitulates all dysregulated immune and barrier functions of human AD skin, OVA-challenged mice replicate activation of inflammatory pathways observed in patients with AD. Moreover, OVA as an AD-inducing agent can be used on different mouse backgrounds to account for genetic variability allowing to identify key players of cutaneous inflammation [14]. In gender-matched experiments, we noticed that male mice do not feature the same histopathological patterns seen in female mice. Pre-lesional skin changes occurring in males after one OVA exposure include hypodermal cell infiltration without epidermal or dermal thickening, suggesting a difference in the nature or the distribution of infiltrating cells between the two sexes. One limitation of our study is that we did not identify the cell types that were increased in the hypodermis. However, reports in mice identified eosinophils, MC and CD3+CD4+ cells [27] and human skin samples featured CD3+ T-cells and CD11c+ dendritic cells in non-lesional skin from patients with AD [28]. Skin changes occurring over time featured gradual chemokine increase, including CCL3 in female Balb/c mice after OVA treatment [27], in agreement with our data [18]. The paucity of reports analyzing the sequence of skin alterations at the onset of AD mouse models in males is noteworthy. Local CCL2, CCL3 and CCL5 mRNA and CCL2 and CCL3 protein levels were elevated in female mice [18] but not in male mice (our current study). These chemokines have been shown to induce recruitment of macrophages, granulocytes, CD3+ and CD4+ cells [23,29]. Importantly, MC secrete these chemokines [30] and our group previously reported that S1P-mediated MC activation is essential to their local production in early allergic responses of the lung [23] and the skin [18]. In humans, data is mainly collected using skin samples from patients affected with moderate-to-severe AD, with overt skin lesions. Interestingly, many inflammatory chemokines, including CCL2, CCL3 and CCL5 are associated with human chronic, supporting leukocyte recruitment [31]. More recently, seminal studies focusing on the analysis of nonlesional skin specimens collected from patients with lesional AD, also demonstrated an increased inflammatory T cell infiltration, with elevated CCL5, CCL11, CCL17, CCL18 and CCL22 [28]. However, the authors acknowledged that their dataset does not reveal the key chemokines involved in primary pathogenesis of AD, which our studies are focusing on. Using immunohistochemistry, Su et al. established the chemokine profiles to study the evolution of T cell subsets in skin biopsies of 48 patients and their association with the progression of human AD from acute to chronic disease [32]. CCL5 was associated with sub-acute AD. Our current study established that male skin MC are not activated after a single OVA exposure, further supporting that MC activation does promote pre-lesional skin remodeling through local chemokine elevation. It has been postulated that increased local chemokine levels may result in systemic dissemination and elevated serum levels [33]. However, male mice did not display any elevation of local chemokines despite augmented serum levels. Brunner et al. suggested the occurrence of a mutual regulation with correlation of blood and skin markers in lesional and nonlesional human AD skin [34]. In this study, chemokines shared concomitant regulation between *chronic* lesional skin and serum in patients with moderate-to-severe AD. The authors found a strong systemic inflammation with elevated skin chemokine levels in this cohort of overweight patients, a plausible explanation for a pre-existing systemic inflammatory state. Because AD leads to systemic inflammation, Lauffer et al. proposed to use serum levels of chemokines, including CCL2, CCL3 and CCL5, as possible predictors of AD persistence in children [35]. However, no data was provided pertaining to skin-associated chemokine levels. Our data suggests that systemic detection of chemokines may perhaps mark resistance to AD development whereas local elevation may denote AD onset, susceptibility or a sign of active disease. Little is known about the dynamics of chemokine production in serum and in skin tissue or about the relative contributions of skin resident cells to inflammation development during the pre-lesional phase of AD, which both warrant further investigation.

It is noteworthy that serum IgE levels are elevated in ~80% of patients with AD however, IgE levels do not necessarily correlate with disease severity [16]. In male mice, IgE levels were significantly elevated after 3 OVA patches, thus validating this AD model for both genders, although three skin exposures to OVA induced 2.5 times more serum IgE in female [18] compared to male mice (Figure 3a). Similar to our findings in female mice and as expected, a single OVA skin exposure did not suffice to elevate serum IgE levels in male mice (Figure 3a).

Previous reports have demonstrated that epidermal thickening is observed in AD lesions with or without the presence of MC in filaggrin-deficient mice, rendering MC dispensable in this genetic model [36]. Notably, the disruption of the epidermal barrier due to filaggrin deficiency preceded any local MC activation in this model. It is tempting to speculate that MC may further perturb the skin barrier to enable AD. In contrast, other reports concluded that MC were protective to the epidermal barrier, also suggesting that MC mediators regulated skin barrier integrity [37]. However, the mouse gender was not revealed in their study where transgenic rather than allergen-induced AD models were employed, perhaps contributing to MC function discrepancies. Indeed, AD lesions feature neo-angiogenesis with MC-associated vascular endothelial growth factor-A (VEGF) [38]. In human AD lesions, epidermal thickening is observed, independently of MC localization to the epidermis [39]. In our current study, increased cellular infiltrate in the hypodermis and around BV was seen, as in female mice [18], but the absence of male skin remodeling was linked to lack of MC activation and of local CCL2, CCL3 and CCL5 mRNA and proteins.

We previously identified a novel signaling pathway in MC by which SphK1-produced S1P from substrate sphingosine binds S1P receptor 2 (S1PR2) [23,39-43]. signaling through signal transducer and activator of transcription 3 to release CCL2, CCL3, CCL5, resulting in T-cell recruitment [23]. S1P/S1PR2 signaling in MC also triggered VEGF and matrix metalloproteinase 2 release, further pointing to an important role for MC in pre-lesional AD skin remodeling [44]. However, the MC/S1P axis was not disease inducing in male mice using the OVA-induced AD model, as skin levels of sphingosine, S1P or mRNA coding for SphK1 were not elevated. Moreover, MC degranulation was not augmented after OVA compared to control male mice, further supporting a critical role for S1P-mediated MC activation in pre-lesional AD skin remodeling, as observed in female mice. Nonetheless, OVA exposure did increase hypodermal cellular infiltration and systemic chemokine levels in male mice and three OVA exposures resulted in elevated serum IgE. Together, our results suggest that male mice may undergo an attenuated and/or delayed skin inflammation due to the absence of MC activation. Indeed, previous studies suggest that female sex hormones may exacerbate Th2 inflammation. Subcutaneous estradiol injection initiates allergic sensitization [45] and estradiol promotes *in vitro* MC degranulation [46,47]. Anaphylactic responses are also more severe in female than male mice [48].

There are potential limitations to this study. First, we are well aware that the mechanisms that underlie sex-related differences in skin disease and inflammation, while, mostly unknown, have not been addressed here. In addition to sex hormones as discussed above, genetic background and differences in skin barrier properties may also contribute to these differences. A better understanding of sex-related differences in skin inflammation may help better prevent, diagnose, and treat inflammatory skin diseases. Second, while we previously demonstrated an essential role for SphK1 in the upregulation of skin S1P levels after stimulation [18], SphK1 was similarly yet only assessed transcriptionally. Nonetheless, the unaltered levels of skin S1P after stimulation strongly supported an essential role for SphK1 in S1P elevation over any other isoform of SphK. Collectively, the current study demonstrates contrasted pathogenic mechanisms enabling skin inflammation in preclinical AD in male mice, compared to reports using female mice [18,19].

Clinical evidence had demonstrated that anti-IgE biologics do not affect the course of AD [16,49], but may have therapeutic potential at high doses [50]. Thus, identifying S1P as a key MC stimulus of pre-lesional skin remodeling may be an important milestone in our understanding of factors that govern the transition from healthy to AD-prone skin. Estrogen exerts immunomodulatory functions on IgE-independent MC degranulation and chemokine production and higher susceptibility of human and mouse females to multiple sclerosis is due to higher production of S1PR2 compared to males [10], further supporting important inflammatory disease enabling functions of the MC/S1P axis.

Our data suggests that S1P-mediated activation of MC is a critical event in early-stage AD, absent in male mice, perhaps relevant to higher prevalence of AD in females, supporting MC intervention to regulate the magnitude and/or perhaps time course [51] of inflammatory skin responses in a gender-dependent manner.

## Abbreviations:

AD: atopic dermatitis
OVA: ovalbumin
MCs: mast cells
S1P: sphingosine-1-phosphate
BV: blood vessels

## References

[1] Hanifin JM, Reed ML (2007) A population-based survey of eczema prevalence in the United States. Dermatitis 18: 82–91. 17498413 10.2310/6620.2007.06034

[2] Silverberg JI (2017) Public health burden and epidemiology of atopic dermatitis. Dermatol Clin 35: 283–289. 28577797 10.1016/j.det.2017.02.002

[3] Thyssen JP, Johansen JD, Linneberg A, (2010) The epidemiology of hand eczema in the general population—prevalence and main findings. Contact Dermatitis 62: 75–87. 20136890 10.1111/j.1600-0536.2009.01669.x

[4] Harrop J, Chinn S, Verlato G, (2007) Eczema, atopy and allergen exposure in adults: a population-based study. Clin Exp Allergy 37: 526–535. 17430349 10.1111/j.1365-2222.2007.02679.x

[5] Chen W, Mempel M, Schober W, (2008) Gender difference, sex hormones, and immediate type hypersensitivity reactions. Allergy 63: 418–1427. 18925878 10.1111/j.1398-9995.2008.01880.x

[6] Lee JH, Haselkorn T, Chipps BE, (2006) Gender differences in IgE-mediated allergic asthma in the epidemiology and natural history of asthma: outcomes and treatment regimens (TENOR) study. J Asthma 43: 179–184. 16754518 10.1080/02770900600566405

[7] Simpson CR, Newton J, Hippisley-Cox J, (2009) Trends in the epidemiology and prescribing of medication for eczema in England. J Roy Soc Med 102: 108–117. 19297652 10.1258/jrsm.2009.080211 PMC2746851

[8] Osman M, Hansell AL, Simpson CR, (2007) Gender-specific presentations for asthma, allergic rhinitis and eczema in primary care. Prim Care Respir J 16: 28–35. 17297524 10.3132/pcrj.2007.00006 PMC6634172

[9] Silverberg JI, Hanifin JM (2013) Adult eczema prevalence and associations with asthma and other health and demographic factors: a US population-based study. J Allergy Clin Immun 132: 1132–1138. 24094544 10.1016/j.jaci.2013.08.031

[10] Ridolo E, Incorvaia C, Martignago I, (2019) Sex in respiratory and skin allergies. Clin Rev Allerg Immu 56: 322–332. 10.1007/s12016-017-8661-0 29306980

[11] Fuxench ZCC, Block JK, Boguniewicz M, (2019) Atopic dermatitis in America study: a cross-sectional study examining the prevalence and disease burden of atopic dermatitis in the US adult population. J Invest Dermatol 139: 583–590. 30389491 10.1016/j.jid.2018.08.028

[12] Sacotte R, Silverberg JI (2018) Epidemiology of adult atopic dermatitis. Clin Dermatol 36: 595–605. 30217272 10.1016/j.clindermatol.2018.05.007

[13] Gutermuth J, Ollert M, Ring J, (2004) Mouse models of atopic eczema critically evaluated. Int Arch Allergy Imm 135: 262–276. 10.1159/000082099 15542938

[14] Ewald DA, Noda S, Oliva M, (2017) Major differences between human atopic dermatitis and murine models, as determined by using global transcriptomic profiling. J Allergy Clin Immun 139: 562–571. 27702671 10.1016/j.jaci.2016.08.029

[15] Yoshihisa Y, Andoh T, Matsunaga K, (2016) Efficacy of astaxanthin for the treatment of atopic dermatitis in a murine model. PLoS One 11: e0152288. 27023003 10.1371/journal.pone.0152288 PMC4811408

[16] Brunner PM, Guttman-Yassky E, (2017) The immunology of atopic dermatitis and its reversibility with broad-spectrum and targeted therapies. J Allergy Clin Immun 139: S65–S76. 28390479 10.1016/j.jaci.2017.01.011 PMC5405702

[17] Mu Z, Zhao Y, Liu X, (2014) Molecular biology of atopic dermatitis. Clin Rev Allerg Immu 47: 193–218. 10.1007/s12016-014-8415-1 24715253

[18] Wedman PA, Aladhami A, Chumanevich AP, (2018) Mast cells and sphingosine-1-phosphate underlie prelesional remodeling in a mouse model of eczema. Allergy 73:405–415. 28905998 10.1111/all.13310 PMC10127444

[19] Akdis CA, Bousquet J, Grattan CE, (2019) Highlights and recent developments in skin allergy and related diseases in EAACI journals (2018). Clin Transl Allergy 9: 60. 31832141 10.1186/s13601-019-0299-y PMC6864939

[20] Spergel JM, Mizoguchi E, Brewer JP, (1998) Epicutaneous sensitization with protein antigen induces localized allergic dermatitis and hyperresponsiveness to methacholine after single exposure to aerosolized antigen in mice. J Clin Invest 101: 1614–1622. 9541491 10.1172/JCI1647 PMC508742

[21] Wolters PJ, Mallen-St Clair J, Lewis CC, (2005) Tissue-selective mast cell reconstitution and differential lung gene expression in mast cell-deficient Kit(W-sh)/Kit(W-sh) sash mice. Clin Exp Allergy 35: 82–88. 15649271 10.1111/j.1365-2222.2005.02136.x PMC2271075

[22] Wedman P, Aladhami A, Beste M, (2015) A new image analysis method based on morphometric and fractal parameters for rapid evaluation of in situ mammalian mast cell status. Microsc Microanal 21: 1573–1581. 26492872 10.1017/S1431927615015342 PMC10127439

[23] Oskeritzian CA, Hait NC, Wedman P, (2015) The sphingosine-1-phosphate/sphingosine-1-phosphate receptor 2 axis regulates early airway T-cell infiltration in murine mast cell-dependent acute allergic responses. J Allergy Clin Immun 135: 1008. 25512083 10.1016/j.jaci.2014.10.044 PMC4388821

[24] Odhiambo JA, Williams HC, Clayton TO, (2009) Global variations in prevalence of eczema symptoms in children from ISAAC Phase Three. J Allergy Clin Immun 124: 1251–1258. 20004783 10.1016/j.jaci.2009.10.009

[25] Pesce G, Marcon A, Carosso A, (2015) Adult eczema in Italy: prevalence and associations with environmental factors. J Eur Acad Dermatol 29: 1180–1187. 10.1111/jdv.12784 25363318

[26] Sandstrom MH, Faergemann J (2004) Prognosis and prognostic factors in adult patients with atopic dermatitis: a long-term follow-up questionnaire study. Brit J Dermatol 150: 103–110. 14746623 10.1111/j.1365-2133.2004.05711.x

[27] Wang G, Savinko T, Wolff H, (2007) Repeated epicutaneous exposures to ovalbumin progressively induce atopic dermatitis-like skin lesions in mice. Clin Exp Allergy 37: 151–161. 17210053 10.1111/j.1365-2222.2006.02621.x

[28] Suárez-Fariñas M, Tintle SJ, Shemer A, (2011) Nonlesional atopic dermatitis skin is characterized by broad terminal differentiation defects and variable immune abnormalities. J Allergy Clin Immun 127: 954–964. 21388663 10.1016/j.jaci.2010.12.1124 PMC3128983

[29] Gaga M, Ong YE, Benyahia F, (2008) Skin reactivity and local cell recruitment in human atopic and nonatopic subjects by CCL2/MCP-1 and CCL3/MIP-1alpha. Allergy 63: 703–711. 18070228 10.1111/j.1398-9995.2007.01578.x

[30] Mukai K, Tsai T, Saito H, (2018) Mast cells as sources of cytokines, chemokines, and growth factors. Immunol Rev 282: 121–150. 29431212 10.1111/imr.12634 PMC5813811

[31] Homey B, Steinhoff M, Ruzicka T, (2006) Cytokines and chemokines orchestrate atopic skin inflammation. J Allergy Clin Immun 118: 178–189. 16815153 10.1016/j.jaci.2006.03.047

[32] Su C, Yang T, Wu Z, (2017) Differentiation of T-helper cells in distinct phases of atopic dermatitis involves Th1/Th2 and Th17/Treg. Eur J Inflammation 15: 46–52.

[33] Ungar B, Garcet S, Gonzalez J, (2017) An integrated model of atopic dermatitis biomarkers highlights the systemic nature of the disease. J Invest Dermatol 137: 603–613. 27825969 10.1016/j.jid.2016.09.037

[34] Brunner PM, Suárez-Fariñas M, He H, (2017) The atopic dermatitis blood signature is characterized by increases in inflammatory and cardiovascular risk proteins. Sci Rep 7: 8707. 28821884 10.1038/s41598-017-09207-z PMC5562859

[35] Lauffer F, Baghin V, Standl M, (2021) Predicting persistence of atopic dermatitis in children using clinical attributes and serum proteins. Allergy 76: 1158–1172. 32794228 10.1111/all.14557

[36] Sulcova J, Meyer M, Guiducci E, (2015) Mast cells are dispensable in a genetic mouse model of chronic dermatitis. Am J Pathol 185: 1575–1587. 25843682 10.1016/j.ajpath.2015.02.005

[37] Sehra S, Serezani APM, Ocaña JA, (2016) Mast cells regulate epidermal barrier function and the development of allergic skin inflammation. J Invest Dermatol 136: 1429–1437. 27021404 10.1016/j.jid.2016.03.019 PMC4921316

[38] Groneberg DA, Bester C, Grützkau A, (2005) Mast cells and vasculature in atopic dermatitis-potential stimulus of neoangiogenesis. Allergy 60: 90–97. 15575937 10.1111/j.1398-9995.2004.00628.x

[39] Oskeritzian CA, Milstien S, Spiegel S (2007) Sphingosine-1-phosphate in allergic responses, asthma and anaphylaxis. Pharmacol Therapeut 115: 390–399. 10.1016/j.pharmthera.2007.05.011 PMC2082108 17669501

[40] Price MM, Oskeritzian CA, Milstien S, (2008) Sphingosine-1-phosphate synthesis and functions in mast cells. Future Lipidol 3: 665–674. 19802381 10.2217/17460875.3.6.665 PMC2749270

[41] Oskeritzian CA, Alvarez SE, Hait NC, (2008) Distinct roles of sphingosine kinases 1 and 2 in human mast cell functions. Blood 111: 4193–4200. 18178871 10.1182/blood-2007-09-115451 PMC2971746

[42] Oskeritzian CA, Price MM, Hait NC, (2010) Essential roles of sphingosine-1-phosphate receptor 2 in human mast cell activation, anaphylaxis, and pulmonary edema. J Exp Med 207: 465–474. 20194630 10.1084/jem.20091513 PMC2839150

[43] Oskeritzian CA (2015) Mast cell plasticity and sphingosine-1-phosphate in immunity, inflammation and cancer. Mol Immunol 63: 104–112. 24766823 10.1016/j.molimm.2014.03.018 PMC4226394

[44] Chumanevich A, Wedman P, Oskeritzian CA (2016) Sphingosine-1-phosphate/sphingosine-1-phosphate receptor 2 axis can promote mouse and human primary mast cell angiogenic potential through upregulation of vascular endothelial growth factor-A and matrix metalloproteinase-2. Mediat Inflamm 2016: 1503206. 10.1155/2016/1503206 PMC4738939 26884643

[45] Holt PG, Britten D, Sedgwick JD (1987) Suppression of IgE responses by antigen inhalation: studies on the role of genetic and environmental factors. Immunology 60: 97–102. 3817869 PMC1453366

[46] Zaitsu M, Narita SI, Lambert KC, (2007) Estradiol activates mast cells via a non-genomic estrogen receptor-alpha and calcium influx. Mol Immunol 44: 1977–1985. 17084457 10.1016/j.molimm.2006.09.030 PMC2603032

[47] Vliagoftis H, Dimitriadou V, Boucher W, (1992) Estradiol augments while tamoxifen inhibits rat mast cell secretion. Int Arch Allergy Imm 98: 398–409. 10.1159/000236217 1384869

[48] Hox V, Desai A, Bandara G, (2015) Estrogen increases the severity of anaphylaxis in female mice through enhanced endothelial nitric oxide synthase expression and nitric oxide production. J Allergy Clin Immun 135: 729–736. 25553642 10.1016/j.jaci.2014.11.003 PMC5586107

[49] Paller AS, Kabashima K, Bieber T (2017) Therapeutic pipeline for atopic dermatitis: end of the drought? J Allergy Clin Immun 140: 633–643. 28887947 10.1016/j.jaci.2017.07.006

[50] Chan S, Cornelius V, Cro S, (2019) Treatment effects of omalizumab on severe pediatric atopic dermatitis: the ADAPT randomized clinical trail. JAMA Pediatr 174: 29–37. 10.1001/jamapediatrics.2019.4476 PMC6902112 31764962

[51] Cruz-Orengo L, Daniels BP, Dorsey D, (2014) Enhanced sphingosine-1-phosphate receptor 2 expression underlies female CNS autoimmunity susceptibility. J Clin Invest 124: 2571–2584. 24812668 10.1172/JCI73408 PMC4089451

